# Synthetic viability induces resistance to immune checkpoint inhibitors in cancer cells

**DOI:** 10.1038/s41416-023-02404-w

**Published:** 2023-08-24

**Authors:** Mingyue Liu, Qi Dong, Bo Chen, Kaidong Liu, Zhangxiang Zhao, Yuquan Wang, Shuping Zhuang, Huiming Han, Xingyang Shi, Zixin Jin, Yang Hui, Yunyan Gu

**Affiliations:** 1 Department of Systems Biology, College of Bioinformatics Science and Technology, Harbin Medical University, Harbin, China; 2 The Sino-Russian Medical Research Center of Jinan University, The Institute of Chronic Disease of Jinan University, The First Affiliated Hospital of Jinan University, Guangzhou, China; 3 Department of Biochemistry and Molecular Biology, Harbin Medical University, Harbin, China

**Keywords:** Tumour biomarkers, Transcriptomics, Cancer immunotherapy

## Abstract

**Background:**

Immune checkpoint inhibitors (ICI) have revolutionized the treatment for multiple cancers. However, most of patients encounter resistance. Synthetic viability (SV) between genes could induce resistance. In this study, we established SV signature to predict the efficacy of ICI treatment for melanoma.

**Methods:**

We collected features and predicted SV gene pairs by random forest classifier. This work prioritized SV gene pairs based on CRISPR/Cas9 screens. SV gene pairs signature were constructed to predict the response to ICI for melanoma patients.

**Results:**

This study predicted robust SV gene pairs based on 14 features. Filtered by CRISPR/Cas9 screens, we identified 1,861 SV gene pairs, which were also related with prognosis across multiple cancer types. Next, we constructed the six SV pairs signature to predict resistance to ICI for melanoma patients. This study applied the six SV pairs signature to divide melanoma patients into high-risk and low-risk. High-risk melanoma patients were associated with worse response after ICI treatment. Immune landscape analysis revealed that high-risk melanoma patients had lower natural killer cells and CD8^+^ T cells infiltration.

**Conclusions:**

In summary, the 14 features classifier accurately predicted robust SV gene pairs for cancer. The six SV pairs signature could predict resistance to ICI.

## Background

Immune checkpoint inhibitors (ICI) revolutionized the treatment of patients with solid tumors, such as advanced melanoma [1, 2]. ICI enhances T cell activity by inhibiting immunosuppressive checkpoint molecules, including cytotoxic T-lymphocyte associated antigen 4 (CTLA-4), programmed cell death 1 (PD-1), and programmed cell death protein ligand 1 (PD-L1), and so on. Nevertheless, despite the clinical success of ICI, only approximately a third of patients exhibit durable responses but will eventually experience a relapse [3]. Resistance to ICI was evident in clinical trials, where within the cohort of advanced melanoma patients, only 16% achieved a complete response (CR) [4]. Hence, exploring resistant biomarkers for ICI and screening patients who could benefit from ICI have become urgent problems in the field of immunotherapy [5]. Broadly, hot tumors with the characteristic of T cell infiltration, high expression of PD-L1 (encoded by *CD274*), and up-regulation of interferon gamma signatures respond to ICI treatment, while cold tumors not [6–8]. Indeed, tumor mutational burden (TMB) has been shown to be an efficient biomarker of the response to treatment with ICI in solid tumors [9]. However, ICI biomarkers for stratifying responders to immunotherapy differ considerably across various cancer types, the underlying resistant mechanism remained unknown.

Synthetic viability (SV), also known as synthetic rescue, one type of genetic interactions, mediates both primary or adaptive resistance in cancer therapy [10–12]. SV is defined as the combination of gene alterations that can rescue the lethal effects of a single gene alteration [11, 13]. Currently, genome-wide CRISPR/Cas9 screens are increasingly used to identify SV gene pairs and to determine mechanisms of drug resistance [14–16]. However, the roles of SV in cancer cell resistance to ICI are still elusive. Cancer driver genes are classified as oncogenes and tumor suppressor genes (TSGs) [17]. Recently, using CRISPR/Cas9, Parrish et al. identified SV gene pairs of TSGs, such as *CDKN2A*/*CDK2NB* and *FBXO25*/*FBXO32* [18]. Zhao et al. systematically analyzed how combinations of inactivated TSGs altered the tumorigenic growth characteristics [19]. Furthermore, many of the genetic interactions have been identified in a context-specific cell line and may not be reproduced across multiple studies [20, 21]. Therefore, it is urgent to systematically identify robust TSGs and oncogenes related SV gene pairs, which may participate in ICI resistance.

In this study, we developed an ensemble classifier to predict robust cancer genes related SV gene pairs by integrating genome-wide CRISPR/Cas9 screens in cancer cell lines. Then, we investigated the impact of SV gene pairs alterations on prognosis in patients across various cancer types. Moreover, we explored the six SV pairs signature to predict the clinical benefit of ICI treatment for melanoma patients. This study also compared the six SV pairs signature with previously published signatures of response to immunotherapy. Finally, we dissected the role of the immune landscape in determining the benefit of immunotherapy.

## Methods

### Multi-omics data of cancer cell lines and bulk pan-cancer samples

We obtained CRISPR/Cas9 genetic perturbation data, expression, somatic mutation, and gene level copy number data from DepMap Portal (version 21Q4) (https://depmap.org/portal/), which consists of 859 cancer cell lines [22]. Gene expression and copy number alterations (CNA) data of 32 cancer types of The Cancer Genome Atlas (TCGA, https://portal.gdc.cancer.gov/) were downloaded from the UCSC Xena (https://xenabrowser.net/datapsges/). CNA were determined by Genomic Identification of Significant Targets in Cancer (GISTIC) [23]. We also acquired mutation profiles of 32 cancer types from cBioPortal (https://www.cbioportal.org).

### Melanoma cohorts with immune checkpoint inhibitors treatment

This study constructed SV signature to predict immunotherapy response for melanoma patients. The Liu2019 cohort, including 121 melanoma patients, was used as a training cohort. Other datasets were validation cohorts. Expression, mutation, CNA data, and clinical information of immunotherapy melanoma patients were obtained from the cBioPortal (https://www.cbioportal.org) and the literature (Supplementary Table S1). The clinical survival outcomes included overall survival (OS), progression-free survival, and disease-free survival. To define the response and non-response patients, we used the criteria defined in the original clinical trials as much as possible. For the Liu2019 cohort where response evaluation criteria in solid tumors (RECIST) information was available, we used CR or partial response (PR) as response and progressive disease (PD) as non-response [7]. In Gide2019, melanoma patients with CR or PR or stable disease (SD) > six months were classified as responders, while melanoma patients with PD or SD < six months were classified as non-responders based on RECIST [24]. For Riaz2017 cohort, the TMB was obtained from the original study.

### Feature score and classifier construction

Paralog gene pairs were obtained from Kegel et al. [25]. Protein complex membership information were downloaded from the comprehensive resource of mammalian protein complexes database (CORUM, 10.1093/nar/gky973) [26]. Shared protein-protein interactors (PPI), a quantitative feature, estimated the shared PPI of gene_1_ and gene_2_ by applying a hypergeometric test. The “Igraph” R package was used to calculate the average shortest distance. The essentiality of shared PPI was defined as the mean essentiality of shared PPI of gene_1_ and gene_2_. The similarity of biological process score, estimated from “GOSemSim” R package, was defined as the similarity of biological process annotation of gene_1_ and gene_2_ in Gene Ontology. The details information of 14 features was presented in Supplementary Table S2.

Next, we used the random forest to assemble the classifier implementation in “randomForest” R package. To prevent over-fitting, we selected low maximum tree depth and relatively high minimum leaf node weight. The parameters used in the final model were as follows: ntree = 500 (i.e., number of trees), mtry = 3, importance = TRUE, proximity = TRUE. All parameters not listed were left as the default.

TSGs and oncogenes were downloaded from the TSGene and ONGene databases, respectively [27, 28]. We focused on four types of SV gene pairs (Supplementary Fig. S1A): (i) TSG_L_-TSG_L_ interaction, where the loss function of TSG_1_ was rescued by the loss function of TSG_2_ [18]; (ii) OG_L_-OG_G_ interaction, where the loss function of oncogene caused by drug inhibition was rescued by the gain function of another oncogene; (iii) OG_L_-TSG_L_ interaction, where the loss function of oncogene caused by drug inhibition was rescued by the loss function of TSG [11]; (vi) TSG_L_-OG_G_ interaction, where the loss function of TSG was rescued by the gain function of oncogene [29]. Loss or gain function of gene was estimated from gene expression, mutation, and copy number data (see details in Supplementary methods).

### Filtered SV by CRISPR/Cas9 screens

To identify the significant association between gene_2_ alteration (loss or gain of function) and gene_1_ dependency, we used a multiple linear regression model with the form: gene_1__dependency ~ gene_2__status (loss or gain of function) + C (cell_line_lineage) (Supplementary Fig. S1C, left). Cell lines dependency score was downloaded from the DepMap Portal (version 21Q4). Parental cell lines information, expression, mutation, and copy number data were downloaded from the Cancer Cell Line Encyclopedia project (CCLE, https://portals.broadinstitute.org/ccle) [22]. The number of cell lines for each cancer type was presented in Supplementary Fig. S2. We restricted gene_2_ status (loss or gain of function) alteration in more than ten cell lines. We applied *false discovery rate (FDR)* multiple testing correction on the *P* value. Gene pairs with a negative coefficient and *FDR*-adjusted *P* value less than 0.05 were considered as candidate SV.

### Survival analysis in TCGA

The patients were divided into two groups according to the status of SV gene pairs as follows: patients with simultaneous alteration (loss or gain of function) of gene_1_ and gene_2_ and others (Supplementary Fig. S1C, right). The OS time of the two groups for specific cancer types was tested using log-rank test, *P* < 0.05 was considered statistically significant and the results were represented by Kaplan-Meier plots. We defined SV-score as the number of co-occurrence alteration (loss or gain of function) in multi-omics of SV gene pairs in a cancer sample. We defined drug-score for chemotherapy as the number of alteration (loss or gain of function) in multi-omics of SV partners in a cancer sample. The cutoff for high or low SV-score was the median across all samples. We applied multivariate Cox regression analysis to estimate the prognosis of SV-score and drug-score.

### Construction and validation of the SV signature

First, we applied univariate Cox regression analysis to select the six SV gene pairs in immunotherapy melanoma cohorts based on clinical survival-related SV in TCGA skin cutaneous melanoma (SKCM) (Supplementary Fig. S1D). Second, we quantified a risk score for each melanoma patient based on the six SV pairs signature through multivariate Cox regression analysis. The formula of the RiskScore was as follows:

$${{{{{\rm{RiskScore}}}}}}=	\, {{\exp }}[(0.96* {{JUN}}_{{{{{{\rm{L}}}}}}}:{{NR}4A3}_{{{{{{\rm{G}}}}}}})+(0.92* {{CASP}3}_{{{{{{\rm{L}}}}}}}:{{BCL}2L12}_{{{{{{\rm{G}}}}}}})\\ 	+(1.06* {{ETV}6}_{{{{{{\rm{L}}}}}}}:{{FGFR}2}_{{{{{{\rm{G}}}}}}})+(0.98* {{BCL}10}_{{{{{{\rm{L}}}}}}}:{{CBLC}}_{{{{{{\rm{G}}}}}}})\\ 	+ (-1.13* {{EIF}3E}_{{{{{{\rm{L}}}}}}}:{{PIK}3R1}_{{{{{{\rm{L}}}}}}})+(0.98* {{RUNX}1}_{{{{{{\rm{L}}}}}}}:{{LCK}}_{{{{{{\rm{L}}}}}}})]$$

where exp denoted exponential, the SV gene pairs alteration status equaled 1, and the non-alteration status equaled 0. ‘L’ or ‘G’ represented loss or gain of function for gene. “SurvivalROC” R package was used to determine the cutoff for classifying patients into low-risk and high-risk melanoma patients. Finally, the same formula and cutoff were applied to the other validation cohorts and the TCGA SKCM cohort.

### Evaluation of immune infiltration in melanoma samples

A total of 29 immune process signature gene sets were obtained from the study of He et al. and Bagaev et al. [30, 31]. Enrichment scores of the signature gene sets in each sample were determined by applying the single-sample gene set enrichment analysis (ssGSEA) approach of the “GSVA” R package [32]. In the TCGA SKCM cohort, the levels of tumor-infiltrating lymphocytes were evaluated by analyzing the information from Thorsson et al. [33]. The proportions of 22 types of infiltrating immune cells were estimated via the CIBERSORT method based on gene expression data [34].

### Statistical analysis

Functional enrichment analysis and clustering of immune processes were conducted using the “clusterProfler” R package. Associations between the SV gene pairs and OS were presented via the Kaplan-Meier method, survival curves were compared via the log-rank test. C-index was determined to compare the accuracy of the six SV pairs signature with other existing signatures of ICI response [35]. Spearman’s rank correlation analysis was applied to estimate the associations between the six SV pairs signature and other transcriptome signatures. Statistical analysis of comparisons between two groups was conducted using the one-sided Wilcoxon rank sum test. Associations between the six SV pairs signature and immune infiltration, copy number deleted, and gene mutation were analyzed via Fisher’s exact test. The “pROC” R package was used to estimate the area under the curve (AUC) values. R software (https://www.r-project.org/) was applied to perform all statistical analyses. *P* value < 0.05 was considered to indicate significance.

## Results

### Identification of features to predict synthetic viable interactions

**Fig. 1:**
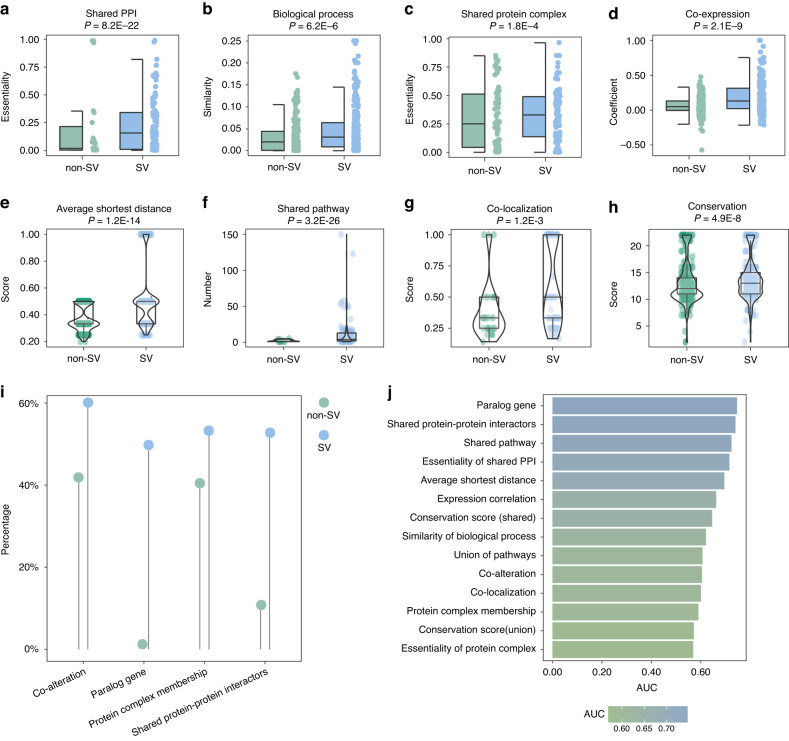
Assessment of the predictive power of features for SV gene pairs. **a**–**h** Significant differences of multiple features between SV and non-SV gene pairs. *P* values were computed by one-sided Wilcoxon rank-sum test and *P* < 0.05 was considered as statistically significant. **i** The percentage of multiple features for SV and non-SV gene pairs. **j** The AUC for each feature as an individual classifier.

We obtained 220 high-confident gene pairs from CRISPR/Cas9 genetic perturbation screens as true-positive SV gene pairs and randomly selected the same number of gene pairs as non-SV. We collected 14 features for SV gene pairs **(**Supplementary Table S2**)**, including comparative properties and boolean (true/false) properties. Comparing with non-SV, SV gene pairs had higher essentiality of shared PPI, biological process similarity score, essentiality of shared protein complex membership, co-expression coefficient, average shortest distance score, pathway number, subcellular co-localization score, and conservation score (*P* = 8.2E-22, *P* = 6.2E-6, *P* = 1.8E-4, *P* = 2.1E-9, *P* = 1.2E-14, *P* = 3.2E-26, *P* = 1.2E-3, *P* = 4.9E-8, one-sided Wilcoxon rank-sum test, Fig. 1a–h). Comparing features between SV and non-SV gene pairs, a high proportion of SV gene pairs had features of co-occurring alteration, paralog gene, protein complex membership and shared PPI (Fig. 1i). We assessed the predictive performance of each feature for SV gene pairs by analyzing receiver-operating characteristic (ROC) and computing AUC. Paralog gene and shared PPI had better performance in predicting SV gene pairs (Fig. 1j). Both paralog gene and shared PPI had an AUC greater than 0.7. Overall, SV gene pairs in cancer cells tended to be functionally similar.

#### An ensemble classifier to predict synthetic viable interactions

**Fig. 2:**
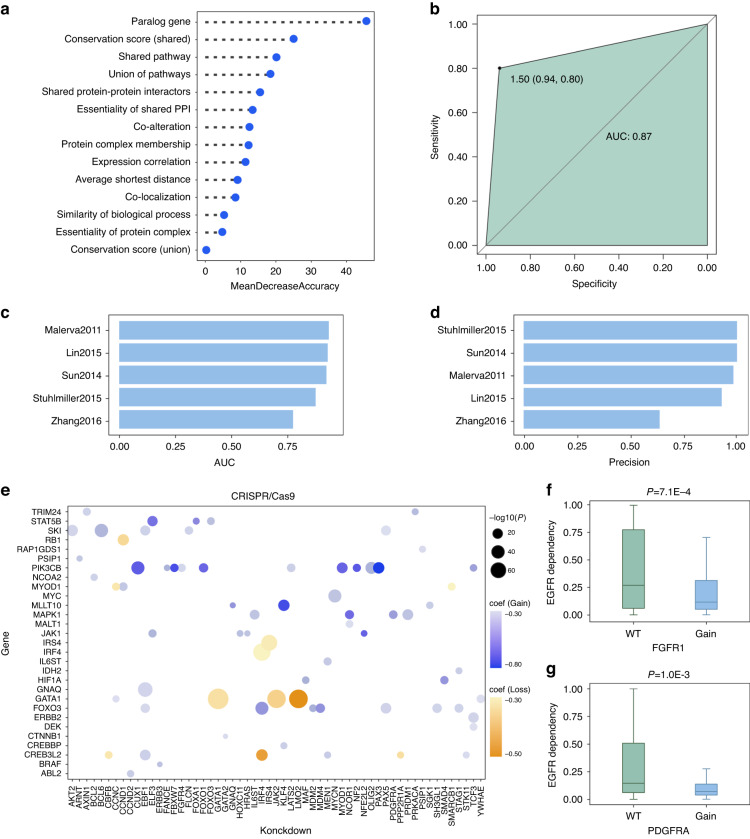
An ensemble random forest classifier to predict SV gene pairs. **a** The weights of 14 features from random forest classifier. **b** ROC curve for ensemble classifier. **c**, **d** The AUC and precision for ensemble classifier’s performance in predicting published resistant genes. **e** The regression coefficients of SV gene pairs were estimated by multiple linear regression model (*FDR*-adjusted *P* < 0.05). Significant differential dependency scores of *EGFR* in cancer cell lines where *FGFR1* (**f**) or *PDGFRA* (**g**) was gain of function versus where *FGFR1* or *PDGFRA* was wild type. *P* values were calculated from the multiple linear regression model and *FDR*-adjusted *P* < 0.05 was considered statistically significant.

This study combined 14 features into a random forest ensemble classifier to predict SV gene pairs. Fig. 2a showed the weights of 14 features in random forest classifier. Random forest classifier achieved an AUC of 0.87 (Fig. 2b). We also investigated classifier performance in predicting the published drug resistant genes (Supplementary Fig. S3). Classifier identified resistant genes with AUCs of 0.75 ~ 1.00 and precision of about 93% (Fig. 2c, d). This work focused on four types of TSGs and oncogenes related SV gene pairs (Supplementary Fig. S1A). We next predicted SV for TSGs and oncogenes-related gene pairs based on 14 features from ensemble random forest classifier (Supplementary Fig. S1B). Then, our study identified 1,861 SV gene pairs using CRISPR/Cas9 genetic perturbation screen, and SV gene pairs with the regression coefficients less than -0.3 were shown in Fig. 2e (*FDR*-adjusted *P* < 0.05, multiple linear regression). Further, we revealed cancer cell lines with gain function of *FGFR1* or *PDGFRA* had lower dependency scores compared to other cancer cell lines, after *EFGR* knockout (*P* = 7.1E-4, Fig. 2f; *P* = 1.0E-3, Fig. 2g, multiple linear regression). In accordance with previous studies, *FGFR1* and *PDGFRA* have been associated with resistance to EGFR inhibitors [36, 37].

#### Prognosis-related SV gene pairs across multiple cancers

**Fig. 3:**
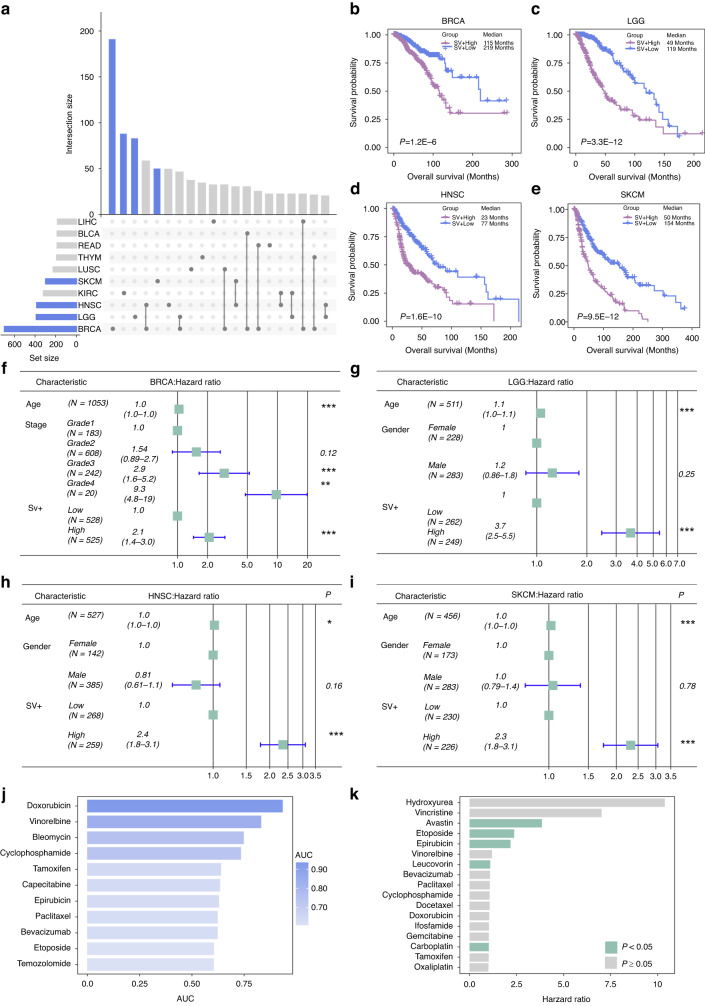
Survival analysis of pan-cancer from TCGA. **a** The overlapping of survival related SV gene pairs across various cancer types. Kaplan-Meier plots of patients with low and high SV-score (cutoff was median of scores) in BRCA (**b**), LGG (**c**), HNSC (**d**), and SKCM (**e**) cohorts. The *P* values were computed by log-rank test. Multivariate Cox regression analysis of the SV-score, age, and stage in BRCA (**f**), LGG (**g**), HNSC (**h**), and SKCM (**i**) cohorts. **j** AUCs for SV gene pairs performance in predicting drug response. **k** Prediction of SV gene pairs in terms of survival: The x-axis displays the hazard ratio of patients result from multivariate Cox regression analysis of SV gene pairs. For *P* value, * denotes *P* < 0.05, ** denotes *P* < 0.01, and *** denotes *P* < 0.001.

We extracted prognosis-related SV gene pairs in the datasets from TCGA (*P* < 0.05, log-rank test, Fig. 3a). In breast carcinoma (BRCA), brain lower grade glioma (LGG), head and neck squamous cell carcinoma (HNSC), and SKCM, patients with high SV-score (defined by median of scores) showed significantly worse OS (*P* = 1.2E-6, Fig. 3b; *P* = 3.3E-12, Fig. 3c; *P* = 1.6E-10, Fig. 3d; *P* = 9.5E-12, Fig. 3e, log-rank test). In the cancer types of BRCA, LGG, HNSC, and SKCM, multivariate Cox regression analysis showed that SV-score was an independent predictive risk factor for prognosis (*P* < 0.05, Fig. 3f–i). Besides, we found that AUCs of predicting response to drug by SV gene pairs were greater than 0.6 for multiple treatments (Fig. 3j). In brief, this work indicated that SV gene pairs were associated with prognosis of patients by multiple treatments (*P* < 0.05, multivariate Cox regression analysis, Fig. 3k).

### Construction of SV signature to predict immunotherapy response for melanoma

This study identified the six SV pairs signature that were significantly related to the prognosis of melanoma with ICI treatment (*P* < 0.05, univariate Cox regression analysis). Next, we quantified a risk score for each melanoma patient based on the six SV pairs signature through multivariate Cox regression model.

**Fig. 4:**
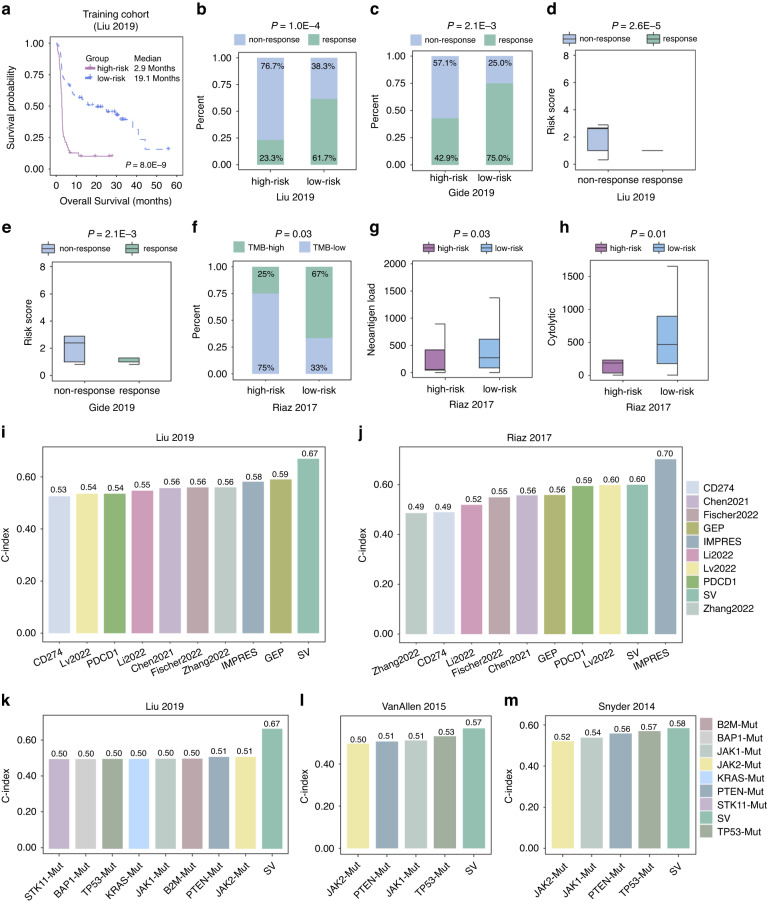
Identification and validation of the SV signatures. **a** Kaplan-Meier plot of melanoma patients with high-risk versus low-risk in training cohort. The *P* values were computed by log-rank test. The proportion of melanoma patients who responded to ICI treatment in the high-risk and low-risk in Liu2019 (**b**) and Gide2019 cohort (**c**). The *P* values were computed by Fisher’s exact test. A significant differential risk score of melanoma patients between response and non-response in Liu2019 (**d**) and Gide2019 (**e**) cohort. The *P* values were computed from one-sided Wilcoxon rank-sum test. **f** The proportion of melanoma with high TMB in the high-risk and low-risk patients in the Riaz2017 cohort. The *P* values were computed by Fisher’s exact test. Significant differential neoantigen load (**g**) and cytolytic score (**h**) of melanoma patients between high-risk and low-risk in Riaz2017 cohort. The *P* values were computed by one-sided Wilcoxon rank-sum test. Comparison of C-indexes between the six SV pairs signature and expression (**i**, **j**) and mutation (**k**, **l**, **m**) associated signatures from published literature across multiple datasets.

We determined a cutoff value (cutoff = 1) to divide melanoma patients into high-risk and low-risk groups. High-risk melanoma patients had shorter OS than low-risk patients in the training cohort (*P* = 8.0E-9, log-rank test, Fig. 4a). Importantly, melanoma patients with high-risk scores were significantly associated with unfavorable response of ICI treatment in the training cohort and validation cohort (*P* = 1.0E-4, Fig. 4b; *P* = 2.1E-3, Fig. 4c, Fisher’s exact test). Patients with ICI non-response exhibited significantly increased risk scores (*P* = 2.6E-5, Fig. 4d; *P* = 2.1E-3, Fig. 4e, one-sided Wilcoxon rank-sum test; *P* = 0.03, Supplementary Fig. S4A; *P* = 0.02, Supplementary Fig. S4B, Kruskal-Wallis test). Low-risk melanoma patients tended to be associated with higher TMB compared with high-risk patients (*P* = 0.03, Fisher’s exact test, Fig. 4f; *P* = 0.02, one-sided Wilcoxon rank-sum test, Supplementary Fig. S4C). Low-risk melanoma patients showed higher neoantigen load and cytolytic score compared with high-risk patients in Riaz2017 cohort (*P* = 0.03, Fig. 4g; *P* = 0.01, Fig. 4h, one-sided Wilcoxon rank-sum test). In addition, this study systematically compared the performance of the six SV pairs signature with the existing signatures of ICI response, such as mutation and expression-associated signatures (Supplementary Table S3). The predictive power of the six SV pairs signature was greater than some existing signatures in Liu2019, Riaz2017, VanAllen2015, and Snyder2014 cohorts (Fig. 4i–m). The six SV pairs signature also presented significant association with some other signatures in multiple cohorts (*P* < 0.05, Spearman’s rank correlation test, Supplementary Fig. S4D).

### Immune landscape of the high-risk and low-risk melanoma patients

**Fig. 5:**
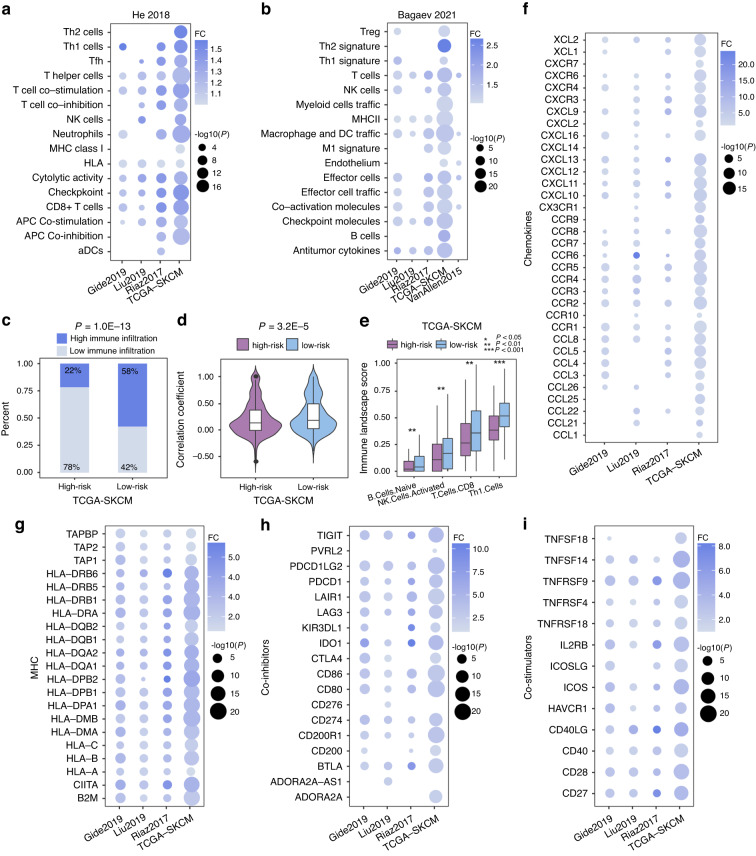
The immune landscape of the high-risk and low-risk patients in multiple melanoma cohorts. Significant differential of 29 immune signatures scores from He et al. (**a**) and Bagaev et al. (**b**) estimated by the ssGSEA method between low-risk and high-risk patients across melanoma cohorts. **c** The proportions of high immune and low immune infiltration estimated by 29 immune signatures from He et al. in the high-risk and low-risk melanoma patients. The *P* values were computed by Fisher’s exact test. **d** Significant differential of correlation coefficients among 29 immune signatures from He et al. between high-risk and low-risk patients in TCGA SKCM dataset. **e** Significant differential immune signatures score from Thorsson et al. between high-risk and low-risk melanoma patients. Significant differential expression of chemokines (**f**), MHC (**g**), co-inhibitors (**h**), and co-stimulators (**i**) genes between low-risk and high-risk patients across melanoma cohorts. The *P* values were calculated from one-sided Wilcoxon rank-sum test with *FDR*-adjusted. Fold change (FC) values were calculated by ratio of mean gene expression from low-risk and high-risk patients. For *P* value, * denotes *P* < 0.05, ** denotes *P* < 0.01, and *** denotes *P* < 0.001.

Using the risk model obtained from the training cohort (see Methods), we classified patients into high-risk and low-risk groups in the immunotherapy melanoma datasets and TCGA SKCM dataset. The low-risk melanoma patients were characterized by greater abundance of natural killer (NK) cells and major histocompatibility complex (MHC) molecules (*FDR*-adjusted *P* < 0.05, one-sided Wilcoxon rank-sum test, Fig. 5a, b, Supplementary Fig. S4E). Meanwhile, we clustered melanoma patients into low-immune and high-immune infiltration groups based on immune signature scores estimated by ssGSEA for 29 immune gene sets from He et al. Interestingly, the high immune cell infiltration samples were significantly enriched in cases from the low-risk samples in TCGA SKCM patients (*P* = 1.0E-13, Fisher’s exact test, Fig. 5c). Additionally, the correlation among immune cell activities in the low-risk patients was significantly higher than that in high-risk patients (*P* < 0.05, Spearman’s rank correlation test, Supplementary Fig. S4F; *P* = 3.2E-5, one-sided Wilcoxon rank-sum test, Fig. 5d). Analyzing the immune landscape score according to Thorsson et al., we found augmented levels of B cells, NK cells, and CD8^+^ T cells in the low-risk patients than high-risk patients (*FDR*-adjusted *P* < 0.05, one-sided Wilcoxon rank-sum test, Fig. 5e). Furthermore, expression of chemokines and MHC genes were higher in the low-risk melanoma patients than that in the high-risk melanoma patients. (*FDR*-adjusted *P* < 0.05, one-sided Wilcoxon rank-sum test, Fig. 5f, g). Immune checkpoint molecules (such as *PDCD1*, *CD274*, and *CTLA4*) and co-stimulatory molecules were also highly expressed in low-risk melanoma group (*FDR*-adjusted *P* < 0.05, one-sided Wilcoxon rank-sum test, Fig. 5h, i). Thus, immune checkpoint molecules may mediate response to ICI treatment in low-risk melanoma patients.

### Genome features between the high-risk and low-risk melanoma patients

**Fig. 6:**
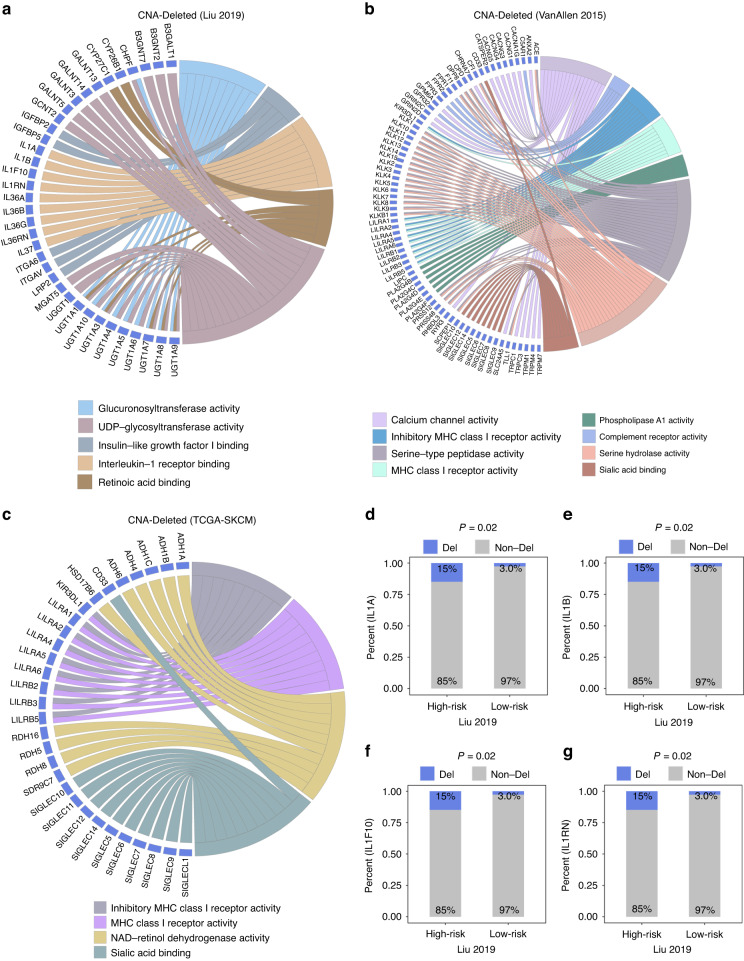
Copy number deleted between high-risk and low-risk patients in the multiple melanoma cohorts. **a**–**c** Copy number deleted genes enrichment with “Molecular Function” in Liu2019, VanAllen2015, and TCGA SKCM cohorts. The proportion of melanoma who occurred copy number deletion of *IL1A* (**d**), *IL1B* (**e**), *IL1F10* (**f**) and *IL1RN* (**g**) in the high-risk and low-risk patients.

This work detected significant differences in mutated genes between the high-risk and low-risk melanoma patients and found that mutated genes were significantly enriched in low-risk patients compared with high-risk patients (*P* < 0.05, Fisher’s exact test, Supplementary Fig. S5A). This result was consistent with the previous discovery that low-risk patients were related to high TMB and neoantigen load (Fig. 4f, g). Copy number deleted genes were significantly enriched in interleukin receptor and MHC class I receptor activity related molecular function in Liu2019, VanAllen2015, and TCGA SKCM cohorts (*P* < 0.05, hypergeometric test, Fig. 6a–c). And, high-risk patients had significant copy number deletion compared with low-risk patients (*P* = 0.02, Fisher’s exact test, Fig. 6d–g). Notably, copy number deleted genes that participating in multiple metabolic-related biological process were significantly enriched in high-risk melanoma patients (*P* < 0.05, hypergeometric test, Supplementary Fig. S5B; *P* = 1.0E-3, *P* = 1.0E-3, *P* = 7.0E-3, *P* = 0.02, Fisher’s exact test, Supplementary Fig. S5C-F). Thus, the results indicated that inactivation of MHC class I and interleukin receptor contributed to resistance to ICI in high-risk melanoma patients.

## Discussion

In this work, we established 14 features ensemble classifier to comprehensively predict robust SV gene pairs for cancer. As excepted, SV gene pairs were related to poor survival across various cancer types in the TCGA. Moreover, this work revealed a signature consisting of the six SV pairs to predict resistance to ICI treatment for melanoma patients. Compared with the low-risk patients, high-risk patients of melanoma showed lower immune cell infiltration of NK cells and CD8^+^ T cell. To the best of our knowledge, this was the first study to investigate SV effect across melanoma cohorts with ICI treatment.

Paralog gene was considered to measure functional similarity for SV gene pairs. Average shortest distance in PPI could also be used to predict SV gene pairs. Genes that have closer distance were more likely to encode similarity functionality, which could explain why gene pairs with closer distance were more likely to be SV. The predictive power of individual feature may be affected by limitations of the underlying data sources, particularly those features derived from PPI and protein localization. The Human Protein Atlas contained high-confidence antibody-derived localization for only part of TSGs and oncogenes. Despite the above-noted limitations, we showed that features were sufficient for learning the general trends that distinguish SV from non-SV gene pairs (Fig. 2b–d).

After constructing the six SV pairs signature to predict ICI response, we calculated overall immune cell infiltration levels for melanoma patients. The immune score was significantly higher in the low-risk patients than in the high-risk patients, which confirmed the stronger anti-tumor immune activity. Many studies have shown that the density of tumor-infiltrating lymphocytes is positively associated with the immune response in kinds of cancers [38]. In addition to a high level of NK cells, B cells, and CD8^+^ T cells, the low-risk patients were characterized by over-expression of immune checkpoint genes, such as *PDCD1*, *CD274*, and *CTLA-4*, compared with the high-risk patients. Therefore, activated anti-tumor immunity, high *PDCD1*, *CD274*, and *CTLA-4* expression, and enhanced tumor immunogenicity might explain why the low-risk melanoma patients were found to be more likely to benefit from ICI treatment than the high-risk patients. Furthermore, we also identified four SV signature that was associated with resistance to ICI treatment in non-small cell lung cancer (Supplementary method, Supplementary Fig. S6).

In summary, this study developed a classifier to predict robust SV gene pairs and systematically identified SV signature for assessing the effect of ICI treatment across multiple melanoma cohorts. Furthermore, our study revealed distinct immune landscapes between high-risk and low-risk melanoma patients. Overall, we proposed a new tumor classification system with the potential to guide ICI treatment decisions. Elucidating the molecular mechanism underlying the effect of each SV gene pairs on immunotherapy in vivo and vitro functional experiments warrant our future work.

### Supplementary information

Supplemental Material

## Data Availability

All data analyzed during this study can be downloaded from TCGA, CCLE, DepMap, and cBioPortal. Other information was provided in the Supplementary Information files.

## Code availability

The code used for the analysis is available upon request. For more detailed information, please contact the corresponding author (YYG).
