# Supplementary material for: Synthetic viability induces resistance to immune checkpoint inhibitors in cancer cells
Source: Br J Cancer. 2023 Aug 24;129(8):1339–49. doi: 10.1038/s41416-023-02404-w (PMC10575993; doi:10.1038/s41416-023-02404-w)
Supplement: Supplementary file 1 — Supplemental Material [file 41416_2023_2404_MOESM1_ESM.docx]

# Synthetic viability induces resistance to immune checkpoint inhibitors in cancer cells

## Supplementary methods

### Definition of loss or gain function of genes

We generated loss or gain function profiles of genes from three types of samples. First, for cell lines from the Cancer Cell Line Encyclopedia project (CCLE, <https://portals.broadinstitute.org/ccle)>, a gene was marked as loss (or gain) function in a given cell line if conformed to at least one of the criteria: (1) log expression value +1 was less than 1 (or greater than 1), (2) log2 copy number ratio was less than -1.28 (or greater than 1.28), (3) loss (or gain) of functional mutation ^1-3^. Specifically, we defined loss (or gain) of functional mutation as gene expression was significantly lower (or higher) in the mutated cell lines compared to wild type cell lines (*P* < 0.05, one-sided Wilcoxon rank-sum test). Second, for tissue samples across multiple cancer types from The Cancer Genome Atlas (TCGA, [https://portal.gdc.cancer.gov/](https://links.jianshu.com/go?to=https://portal.gdc.cancer.gov/)), a gene was marked as loss (or gain) function in a given sample if accorded to at least one of the criteria: (1) gene expression value was less than bottom tertile (or greater than top tertile) across samples in specific cancer type, (2) copy number alterations (CNA) values equal to -1 (or 1), (3) loss (or gain) of functional mutation. For tissue samples, loss (or gain) of functional mutation was defined as gene expression was significantly lower (or higher) in mutated samples compared to wild type samples (*P* < 0.05, one-sided Wilcoxon rank-sum test). Third, for melanoma cohorts treated by immune checkpoint inhibitors (ICI), we only used gene expression data to define loss or gain function of the gene because mutation and CNA information were not available for all samples. A gene was defined as a loss (or gain) of function if the gene expression level was less than the bottom tertile or (greater than the top tertile) across samples in each cohort.

### RiskScore of four SV signature in non-small cell lung cancer

We applied univariate Cox regression analysis to select four SV gene pairs in immunotherapy non-small cell lung cancer (NSCLC) cohorts based on clinical survival related SV in TCGA lung adenocarcinoma and lung squamous cell carcinoma **(Supplementary Fig. S1D)**. Second, we quantified a risk score for each NSCLC patient based on the four SV pairs signature through multivariate Cox regression analysis. The formula of the RiskScore in NSCLC was as follows: RiskScore = exp [(1.22**BCL11B*_L_:*GATA1*_G_) + (1.35**SMARCE1*_L_:*PDGFRA*_G_) + (1.07**FANCG*_L_:*GATA1*_G_) + (1.08**ZBTB16*_L_:*NTRK1*_L_)]. A cutoff value (cutoff = 1) was determined to divide NSCLC into high-risk and low-risk. High-risk NSCLC patients had shorter overall survival and progression-free survival than low-risk NSCLC patients (**Supplementary Fig. S6A-B**). Importantly, NSCLC patients with high-risk scores were significantly associated with resistance to immune checkpoint inhibitors (ICI) treatment in the Ravi2023 cohort (**Supplementary Fig. S6C-D**).

## Supplementary Tables

### **Table S**1. Information of melanoma and NSCLC datasets with ICI treatment.

| **Datasets** | **PMID** | **Transcriptome** | **Genome** | **Drug** | **Drug Response** |
| --- | --- | --- | --- | --- | --- |
| Liu2019 | 31792460 | √ | √ | anti-PD1 inhibitor | √ |
| VanAllen2015 | 26359337 | √ | √ | anti-CTLA4 inhibitor | √ |
| Snyder2014 | 25409260 | √ | √ | anti-CTLA4 inhibitor | √ |
| Gide2019 | 30753825 | √ | × | anti-PD1or anti-PDL1+anti-CTLA4 inhibitor | √ |
| Riaz2017 | 29033130 | √ | √ | anti-PD1 inhibitor | √ |
| Ravi2023 | [37024582](https://pubmed.ncbi.nlm.nih.gov/37024582) | √ | √ | anti-PD1or anti-PDL1 inhibitor | √ |

Table S**2**. Information of 14 features.

| **Feature** | **Description** | **Source** | **AUC** |
| --- | --- | --- | --- |
| Paralog Gene | Whether gene1 and gene2 are paralog genes. | Kegel *et al.*^4^ | 0.75 |
| Shared Protein-Protein Interactors | The -log10 p-value from the hypergeometric test for the shared protein-protein interactors of gene1 and gene2. This captures how significantly gene1 and gene2's interactors overlap. | PathwayCommon^5^ | 0.74 |
| Shared Pathway | The number of pathways that include both gene1 and gene2. | MsigDB | 0.72 |
| Essentiality of Shared PPI | The mean essentiality of gene1 and gene2's shared protein-protein interactors. Essentiality for each interactor is calculated as the percentage of cell lines in which it is essential (CERES score < -0.6). The feature value is 0 when the gene pair has no shared interactors. | PathwayCommon,  DepMap^1^ | 0.72 |
| Average Shortest Distance | 1 is divided by the average shortest distance between gene1 and gene2 in the protein-protein interaction network. | PathwayCommon,  R packages:igraph | 0.7 |
| Expression Correlation | The Spearman correlation coefficient of gene1 and gene2's expression in CCLE. | CCLE | 0.66 |
| Conservation Score (shared) | A number of species, out of twenty, in which both gene1 and gene2 have an ortholog. The twenty species considered are: Mus musculus, Rattus norvegicus, Kluyveromyces lactis, Magnaporthe oryzae, Eremothecium gossypii, Arabidopsis thaliana, Oryza sativa, Schizosaccharomyces pombe, Saccharomyces cerevisiae, Neurospora crassa, Caenorhabditis elegans, Anopheles gambiae, Drosophila melanogaster, Danio rerio, Xenopus (Silurana) tropicalis, Gallus gallus, Macaca mulatta, Pan troglodytes, Canis lupus familiaris, Bos taurus | R packages: homologene | 0.65 |
| Similarity of Biological Process | The similarity of gene1 and gene2's biological process annotation in Gene Ontology. | R packages: GOSemSim | 0.62 |
| Union of Pathways | The number of pathways that include gene1 or gene2. | MsigDB | 0.61 |
| Co-Alteration | The -log10 p-value from the hypergeometric test for the shared alteration cell lines of gene1 and gene2. The alteration profiles are integrated from mutation, CNA, and expression data. The alteration profile columns represent samples, and the rows represent altered genes. | CCLE | 0.6 |
| Co-Localization | The Jaccard index (intersection over union) of gene1 and gene2's subcellular locations. The feature value is positive for gene pairs with known shared localizations. | Human Protein Atlas^6^ | 0.6 |
| Protein Complex Membership | The number of protein complexes that include both gene1 and gene2. | COURM^7^ | 0.59 |
| Conservation Score (union) | A number of species, out of twenty, in which gene1 or gene2 have an ortholog. The twenty species considered are: Mus musculus, Rattus norvegicus, Kluyveromyces lactis, Magnaporthe oryzae, Eremothecium gossypii, Arabidopsis thaliana, Oryza sativa, Schizosaccharomyces pombe, Saccharomyces cerevisiae, Neurospora crassa, Caenorhabditis elegans, Anopheles gambiae, Drosophila melanogaster, Danio rerio, Xenopus (Silurana) tropicalis, Gallus gallus, Macaca mulatta, Pan troglodytes, Canis lupus familiaris, Bos taurus | R packages: homologene | 0.57 |
| Essentiality of Protein Complex | The mean essentiality of the protein complexes in which gene1 and gene2 form subunits. Essentiality for each subunit is calculated as the percentage of cell lines in which it is essential (CERES score < -0.6). The feature value is 0 when neither gene is a protein complex member. | CORUM,  DepMap | 0.57 |

### **Table S3**. The formula of expression-based ICI signature from publisher literature.

| **Author** | **PMID** | **Formula/Gene** |
| --- | --- | --- |
| Fischer2022 | 35174087 | Exp(CD274)+Exp(XPA)+Exp(ERCC4)+Exp(ERCC2) |
| Zhang2022 | 35144208 | (0.17)*Exp(CSF2)+(0.49)*Exp(NKG7)+(-0.11)*Exp(IL24)+(0.18)*Exp(GZMH)+(-0.31)*Exp(CD3D)+(-0.14)*Exp(CTSW)+(-0.12)*Exp(IL11)+(-0.18)*Exp(GZMK)+(-0.26)*Exp(CD3E)+(0.13)*Exp(HLA-DOA)+(-0.3)*Exp(GZMB)+(0.12)*Exp(CXCL1)+(0.13)*Exp(CCL19)+(0.09)*Exp(MMP1)+(0.1)*Exp(SERPINE1)+(-0.072)*Exp(PTHLH)+(-0.09)*Exp(GABRP) |
| Lv2022 | 35088220 | (-0.121)*Exp(SOCS1)+(0.52)*Exp(IL10)+(-0.191)*Exp(CAMK4)+(-0.013)*Exp(CXCL13)+(-0.122)*Exp(KIR2DS4)+(-0.127)*Exp(TESPA1)+(-0.123)*Exp(CD70)+(-0.022)*Exp(ICAM4) |
| Li2022 | 34807232 | Exp(HLA-C)+Exp(HLA-A)+Exp(CD8A)+Exp(NKG7)+Exp(CST7)+Exp(SRGN)+Exp(CALM3)+Exp(PSMB9)+Exp(COTL1)+Exp(LSP1)+Exp(LAG3)+Exp(PDCD1)+Exp(CLIC1)+Exp(CALM2)+Exp(CD74)+Exp(TUBB)+Exp(STMN1)+Exp(TPI1)+Exp(GAPDH)+Exp(HMGB1)+Exp(HMGB2) |
| Chen2021 | 33097495 | (-0.18)*Exp(SFRP4)+(0.29)*Exp(CPXM1)+(-0.23)*Exp(COL5A1) |
| PD1 | [28650338](https://pubmed.ncbi.nlm.nih.gov/28650338/#/_blank) | Exp(PD1) |
| PD-L1 | [25428504](https://pubmed.ncbi.nlm.nih.gov/25428504/#/_blank) | Exp(PD-L1) |
| GEP | [28650338](https://pubmed.ncbi.nlm.nih.gov/28650338/#/_blank) | Exp(CCL5)+Exp(CD27)+Exp(PD-L1)+Exp(CD276)+Exp(CD8A)+Exp(CMKLR1)+Exp(CXCL9)+Exp(CXCR6)+Exp(HLA-DQA1)+Exp(HLA-DRB1)+Exp(HLA-E)+Exp(IDO1)+Exp(LAG3)+Exp(NKG7)+Exp(PDCD1LG2)+Exp(PSMB10)+Exp(STAT1)+Exp(TIGIT) |
| IMPRES | [26997480](https://pubmed.ncbi.nlm.nih.gov/26997480/#/_blank) | PDCD1,OX40L,CD27,CTLA4,CD40,CD28,CD86,CD80,CD137L,PD-L1,VISTA,TIM-3,CD200,CD276,HVEM |

Note: ‘Exp’ denoted gene expression value.

## Supplementary Figures

**
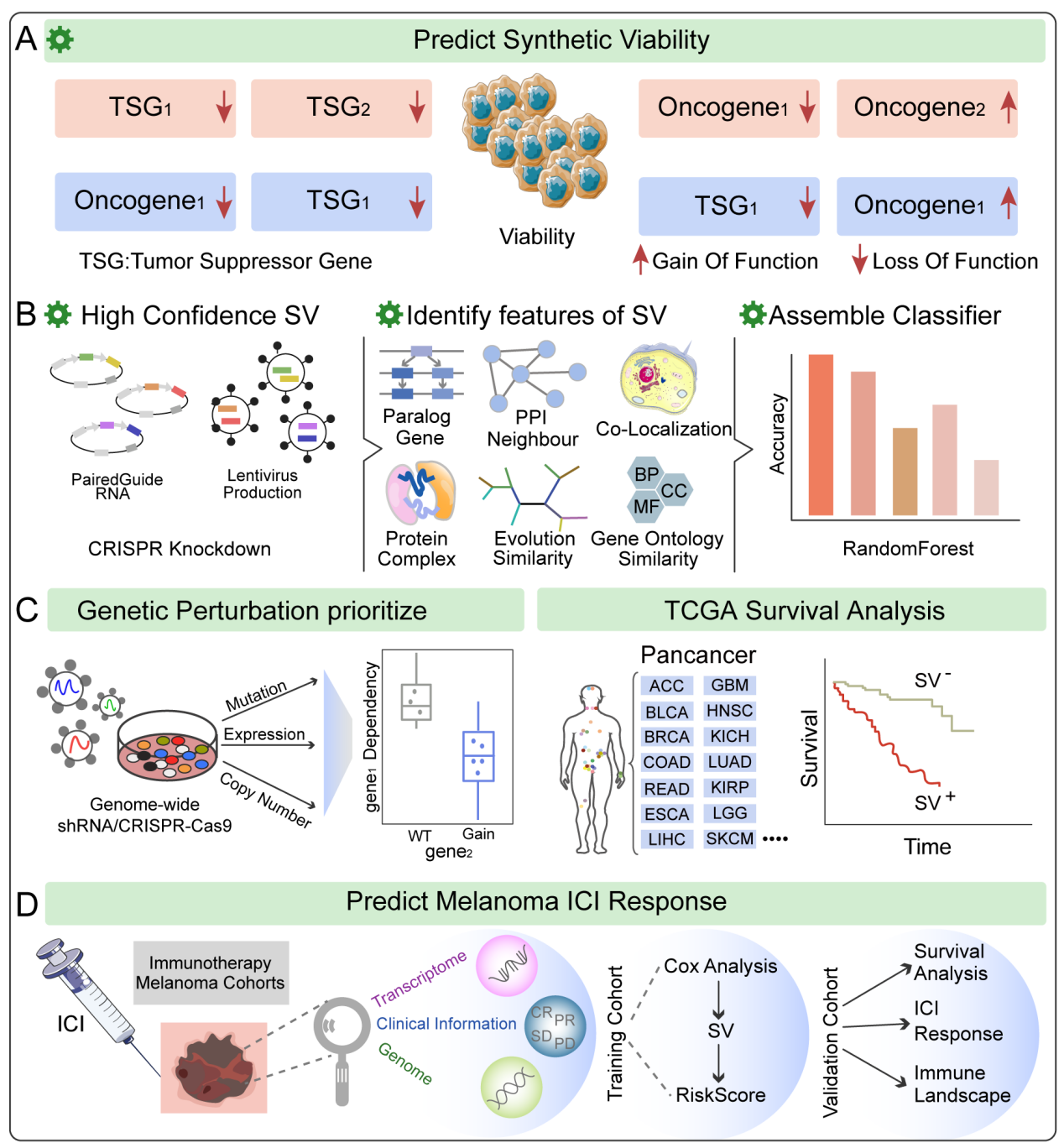
**

**Fig. S1 The flowchart of this work.** (A) Four types of tumor suppressor genes (TSGs) and oncogene related synthetic viability (SV). (B) Collecting highly confident SV, identifying 14 features, and assembling a random forest classifier. (C) Filtering SV in CRISPR/Cas9 screens and extracting prognosis related SV in TCGA patients across various cancers. (D) Identifying and validating ICI response signature in melanoma cohorts.


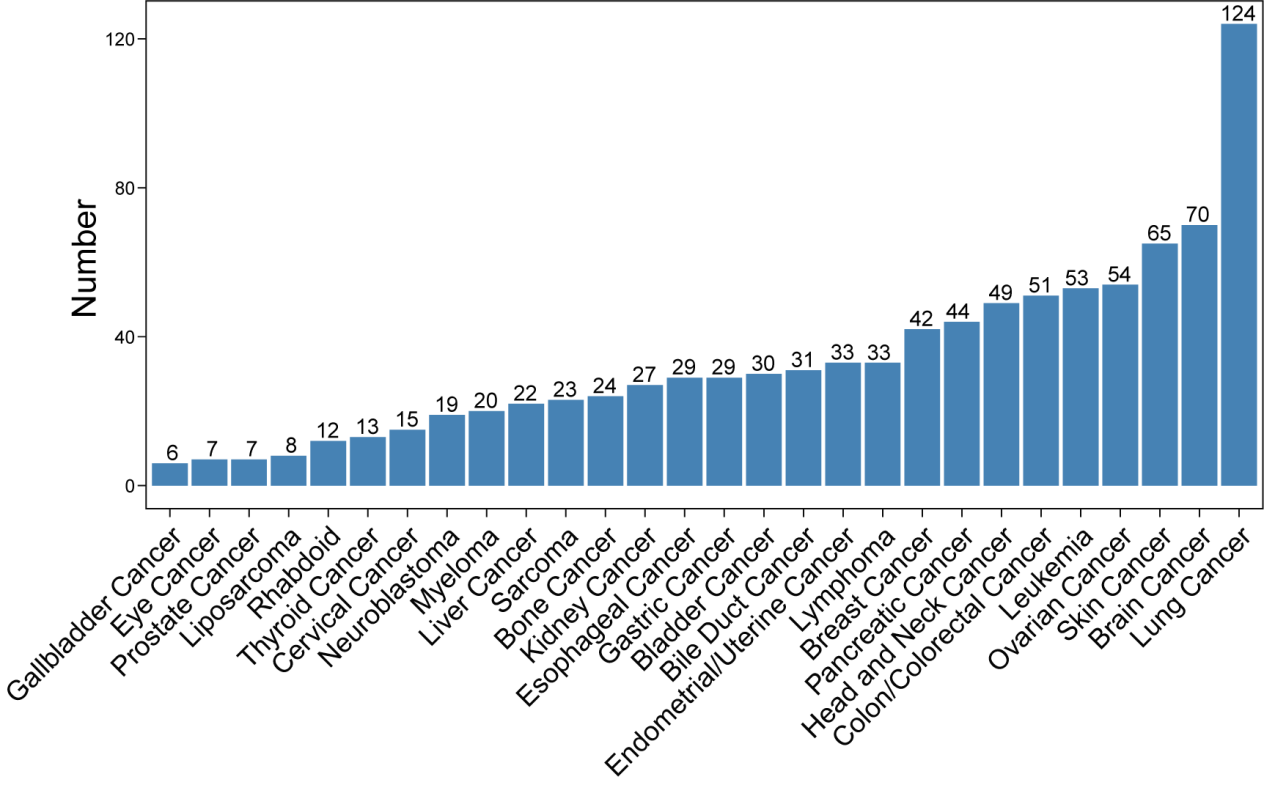


**Fig. S2 The number of cell lines for each cancer type in CRISPR/Cas9 screens.**


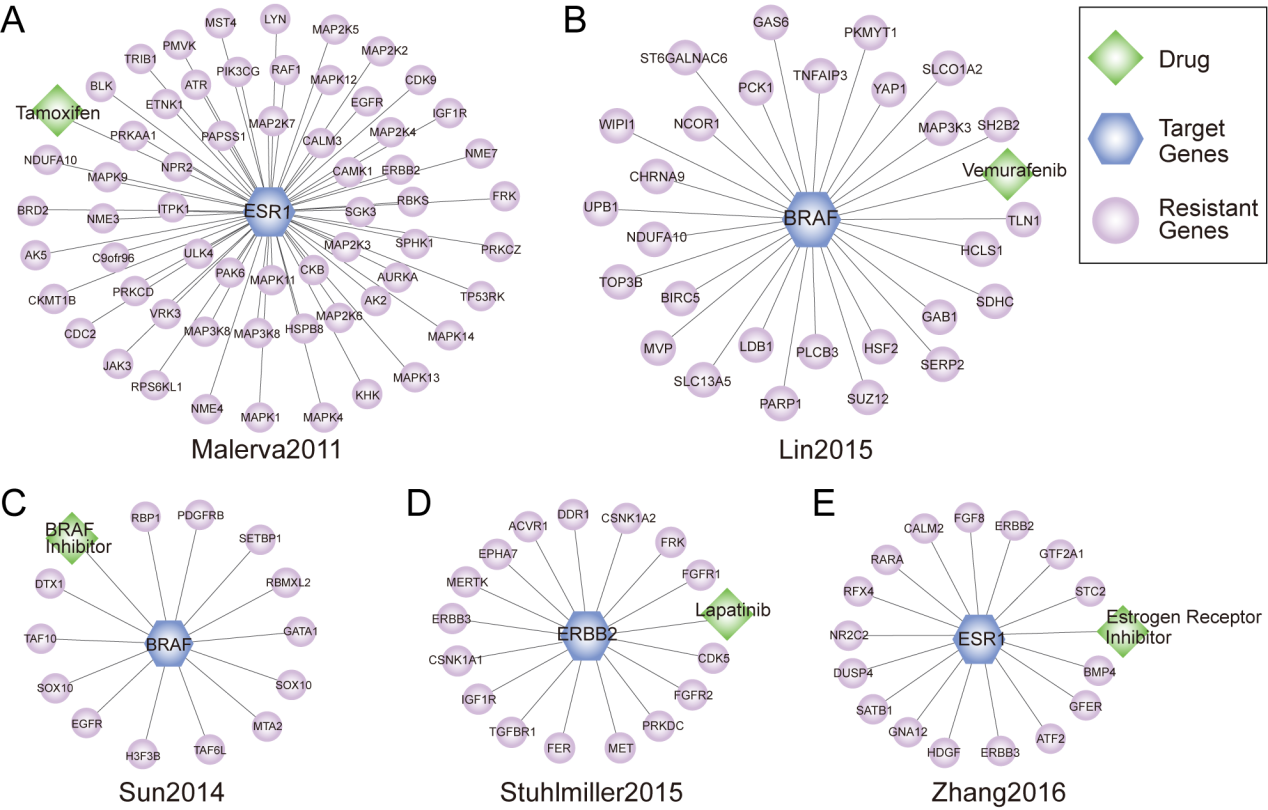


**Fig. S3 The network of drug resistant genes.** (A-E) The network of the drug, target genes, and resistant genes from Malerva2011 (A), Lin2015 (B), Sun2014 (C), Stuhlmiller2015 (D), and Zhang2016 (E). The rhombus nodes represent drugs, hexagon nodes represent the target genes of drugs, and circle nodes represent resistant genes.


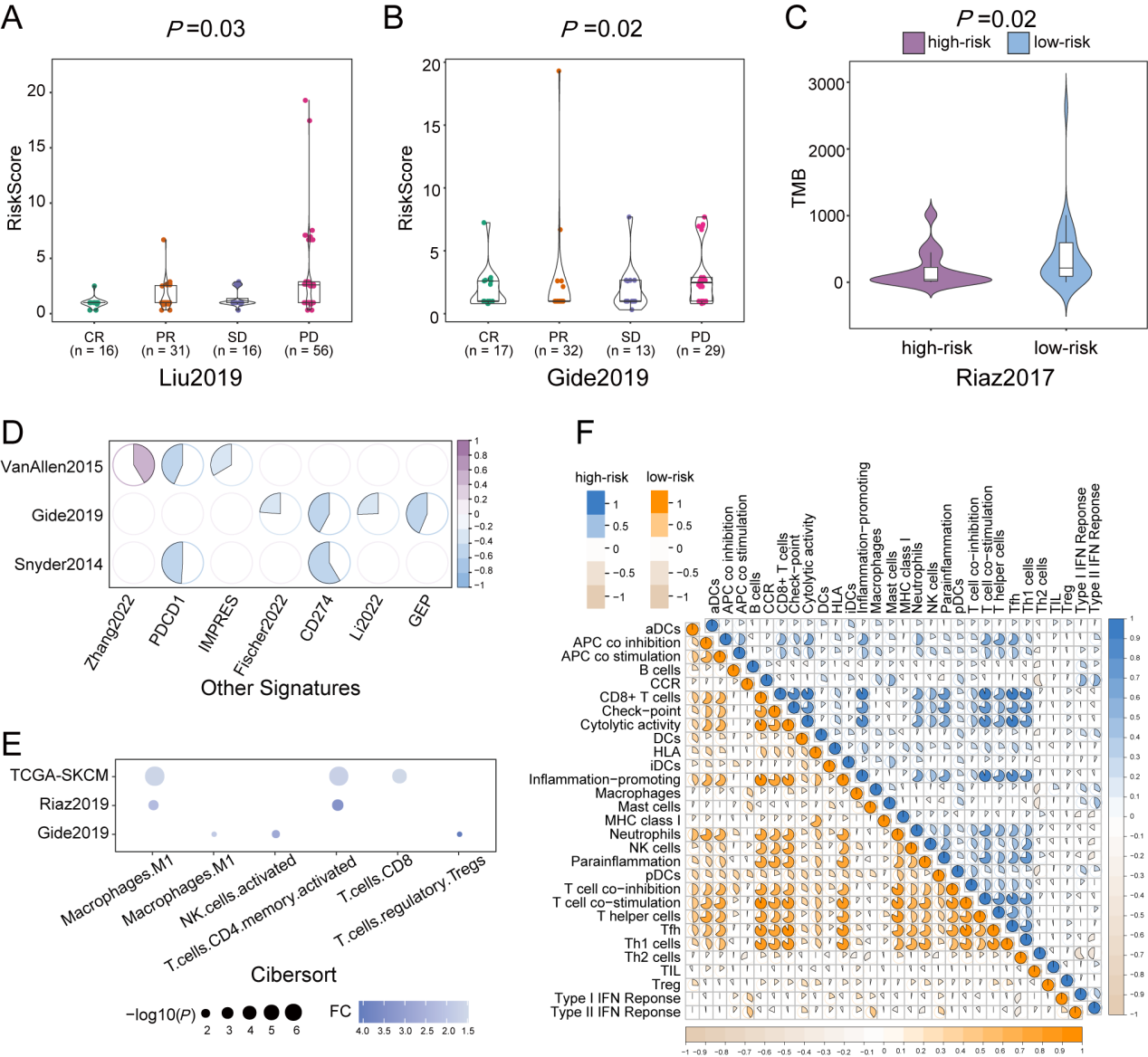


**Fig. S4 Correlation analysis of six SV pairs signature in the melanoma cohorts.** (A-B). The distribution of risk scores in patients with different ICI clinical response status in the training cohort (A) and training cohort (B). The *P* values were computed from the Kruskal-Wallis test. (C) Significant differential tumor mutational burden (TMB) of melanoma between high-risk patients and low-risk patients in Riaz2017 cohort. The *P* values were computed from the one-sided Wilcoxon rank-sum test. (D) Correlation analysis between the six SV pairs signature and other published signatures in VanAllen2015, Gide2019, and Snyder2014 cohorts. (E) Significant differential of CIBERSORT scores between low-risk and high-risk patients across melanoma cohorts. The *P* values were calculated from a one-sided Wilcoxon rank-sum test with *FDR* (*false discovery rate*) correction. FC (fold change) values were calculated by the ratio of mean scores from low-risk and high-risk patients. (F). Correlations among 29 immune process scores estimated from ssGSEA based on He *et al.* immune signature sets in the high-risk (top right panel) and low-risk (low left panel) patients. The correlation coefficient was calculated by Spearman's rank correlation test and *P* < 0.05 was considered as statistical significance.

**
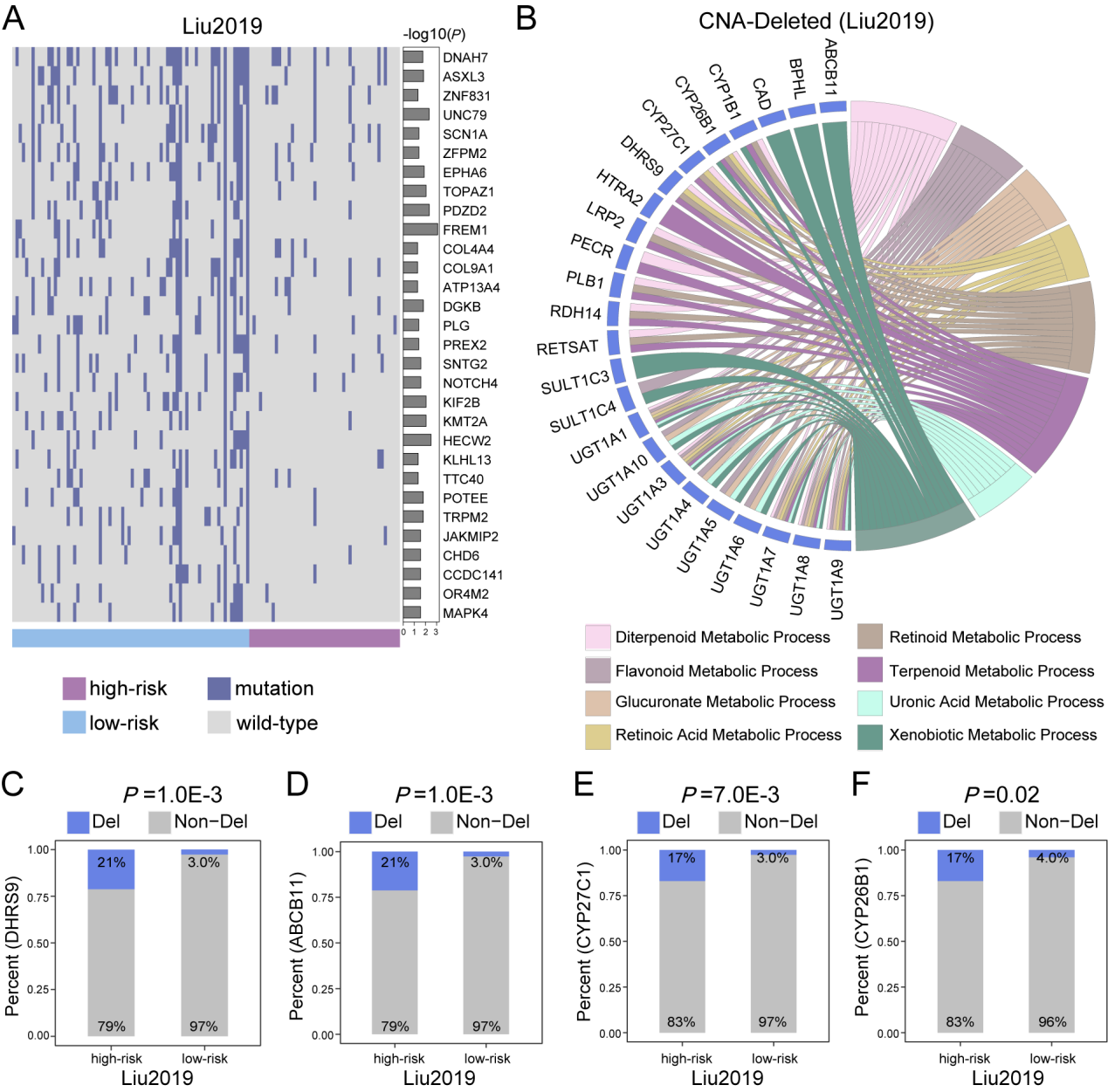
**

**Fig. S5 Somatic mutation and copy number alteration analysis between high-risk and low-risk patients in the melanoma cohorts.** (A). Significant differences in mutated genes between the high-risk and low-risk melanoma patients in the Liu2019 cohort. The *P* values were computed by Fisher's exact test. (B). Copy number deleted genes enrichment in ''Biological Process'' in Liu2019 cohort. (C-F). The proportion of patients with copy number deletion of *DHRS9* (C), *ABCB11* (D), *CYP27C1* (E), and *CYP26B1* (F) in the high-risk and low-risk melanoma patients.

**
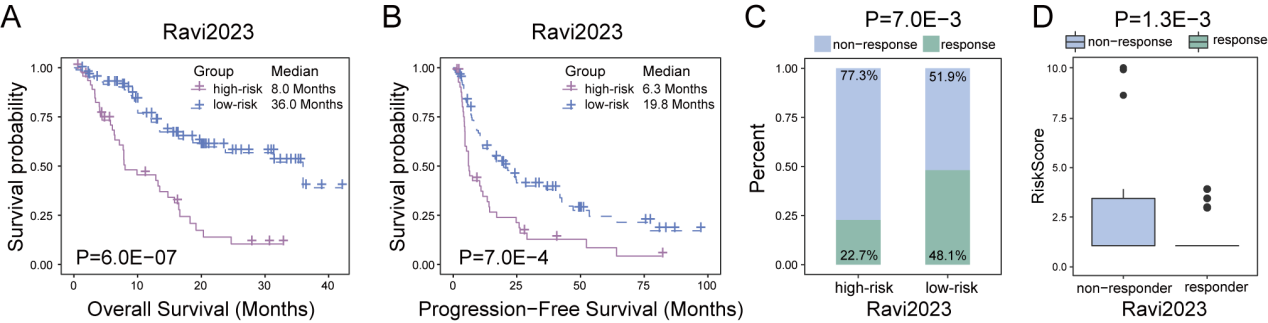
**

**Fig. S6 Identification and validation of four SV signature in NSCLC.** (A-B) Kaplan-Meier plot of NSCLC patients with high-risk versus low-risk in Ravi2023 cohort. The *P* values were calculated by log-rank test. (C) The proportion of NSCLC patients who responded to ICI treatment in the high-risk and low-risk in Ravi2023. The *P* values were calculated by Fisher's exact test. (D) Significant differential risk score of NSCLC patients between response and non-response in Ravi2023 cohort. The *P* values were calculated by one-sided Wilcoxon rank-sum test.

## REFERENCES

1. Ghandi M, Huang FW, Jane-Valbuena J, Kryukov GV, Lo CC, McDonald ER, 3rd, et al. Next-generation characterization of the Cancer Cell Line Encyclopedia. Nature. 2019;569(7757):503-8.

2. Dede M, McLaughlin M, Kim E, Hart T. Multiplex enCas12a screens detect functional buffering among paralogs otherwise masked in monogenic Cas9 knockout screens. Genome Biol. 2020;21(1):262.

3. Nijhawan D, Zack TI, Ren Y, Strickland MR, Lamothe R, Schumacher SE, et al. Cancer vulnerabilities unveiled by genomic loss. Cell. 2012;150(4):842-54.

4. De Kegel B, Quinn N, Thompson NA, Adams DJ, Ryan CJ. Comprehensive prediction of robust synthetic lethality between paralog pairs in cancer cell lines. Cell Syst. 2021;12(12):1144-59 e6.

5. Rodchenkov I, Babur O, Luna A, Aksoy BA, Wong JV, Fong D, et al. Pathway Commons 2019 Update: integration, analysis and exploration of pathway data. Nucleic Acids Res. 2020;48(D1):D489-D97.

6. Digre A, Lindskog C. The Human Protein Atlas-Spatial localization of the human proteome in health and disease. Protein Sci. 2021;30(1):218-33.

7. Ruepp A, Waegele B, Lechner M, Brauner B, Dunger-Kaltenbach I, Fobo G, et al. CORUM: the comprehensive resource of mammalian protein complexes--2009. Nucleic Acids Res. 2010;38(Database issue):D497-501.
